# F96. AGE AND GENDER DETERMINED DIFFERENCES IN THE ONSET OF CHRONIC PHYSICAL MULTIMORBIDITIES AMONG PATIENTS WITH SCHIZOPHRENIA OR DEPRESSION AND THE GENERAL POPULATION

**DOI:** 10.1093/schbul/sby017.627

**Published:** 2018-04-01

**Authors:** Ivona Šimunović Filipčić, Ena Ivezić, Željko Milovac, Ines Kašpar, Sandra Kocijan Lovko, Lada Goršić, Tomislav Gajšak, Strahimir Sučić, Antonija Slaviček Sučić, Majda Grah, Nikolina Tunjić Vukadinović, Žarko Bajić, Marijana Braš, Igor Filipčić

**Affiliations:** 1 University Hospital Center Zagreb; 2 Psychiatric Hospital “Sveti Ivan”

## Abstract

**Background:**

The links between schizophrenia (SCH) or major depressive disorder (MDD) and chronic physical multimorbidities (CPM) are well established. Patients diagnosed with these disorders have a higher prevalence of CPM than the general population (GEP). However, our knowledge of age and gender determined differences in the development of CPM between SCH, MDD, and GEP remains fragmented and inconsistent. This exploratory study intended to compare the onset of CPM in female and male SCH and MDD patients, and the general population (GEP).

**Methods:**

This nested, single-centered, cross-sectional study was performed during 2016 at Psychiatric hospital Sveti Ivan, Zagreb-Croatia. Data were collected for a consecutive sample of 136 patients diagnosed with SCH, 290 diagnosed with MDD, and 861 participants from the general population of the city of Zagreb and Zagreb County. The primary outcome was the prevalence of CPM. The secondary outcome was the prevalence of CPM in the youngest age group ≤35 years.

**Results:**

After adjustment for gender and education, the prevalence of CPM was significantly different between patients with SCH or MDD and GEP (p<0.001). In the oldest age group (≥65 years) the difference was not significant anymore. In the youngest age group, the prevalence was highest in SCH patients (33%) followed by MDD (26%) and GEP (15%) indicating the early onset of CPM in severe mental illness. In the male participants <35 years old, there were no significant differences in the prevalence of CPM between SCH (25%), MDD (23%) and GEP (15%) (p=0.411). However, in the female participants <35 years old the difference was significant and clinically relevant (p=0.006). Prevalence of CPM in female participants was 50% in SCH, 33% in MDD and 14% in GEP.

**Discussion:**

This study finding indicated the earlier onset of CPM in SCH and MDD patients than in GEP. This difference is primarily caused by the high prevalence of CPM in young female patients diagnosed with SCH. More prevalent physical morbidity points to a substantial disadvantage of female SCH patients early in the course of the illness. Understanding the nature and biological basis regarding the risk and outcome of CPM might help to identify new therapeutic targets, allow more individualized treatment, and facilitate better risk prediction and application of healthcare resources.

